# Chain Mediating Role of Illness Perception and Fear of Hypoglycemia in Impaired Hypoglycemic Awareness and Mild Cognitive Impairment in Patients with Type 2 Diabetes Mellitus

**DOI:** 10.2174/0115733998418191251127053922

**Published:** 2026-01-13

**Authors:** Mengyao Han, Xiangning Li, Yuying He, Yinshi Kan, Xiaojuan Wan, Lin Liu, Yue Su, Jing Chen, Li Fang, Jinping Wang, Yu Zhang

**Affiliations:** 1 School of Nursing, Yangzhou University, 136 Jiangyang middle road, Yangzhou City, Jiangsu Province, 225009, China;; 2 Department of Endocrinology, Northern Jiangsu People’s Hospital, Yangzhou, Jiangsu province, China;; 3 Department of Endocrinology, Affiliated Hospital of Yangzhou University, Yangzhou, 225009, China

**Keywords:** Type 2 diabetes mellitus, impaired awareness of hypoglycemia, fear of hypoglycemia, illness perception, mild cognitive impairment, cross-sectional study, mediation analysis

## Abstract

**Introduction:**

Impaired hypoglycemic awareness (IAH) in type 2 diabetes mellitus (T2DM) is linked to mild cognitive impairment (MCI), but the underlying mechanisms are unclear. This study aimed to explore the chain mediating role of two psychological factors, illness perception (IP) and fear of hypoglycemia (FOH), in the relationship between IAH and MCI.

**Methods:**

A cross-sectional survey of 251 T2DM patients was conducted using convenience sampling at two tertiary hospitals. Data on IAH, IP, FOH, and MCI were collected using standardized questionnaires. Data were analyzed and compared using SPSS 27.0 and AMOS 27.0.

**Results:**

There were significant correlations among IAH, IP, FOH, and MCI. Higher IAH was associated with increased FOH and more negative IP, which were both linked to worse cognitive function. Mediation analysis showed that IAH indirectly affects MCI through three pathways: independently *via* FOH, independently *via* IP, and *via* a chained effect of FOH and IP.

**Discussion:**

The findings underscored that the link between IAH and cognitive decline is not solely physiological, but is substantially mediated by psychological responses. Negative illness perceptions and heightened fear, triggered by the unpredictability of IAH, drive maladaptive behaviors and emotional distress that contribute to worse cognitive outcomes.

**Conclusion:**

The findings highlighted the role of psychological factors in cognitive decline among T2DM patients with IAH. We recommend that future interventions consider the associations between these variables. Diverse interventions aimed at improving IP and FOH levels could help slow cognitive decline in patients with IAH.

## 1 INTRODUCTION

As a globally prevalent chronic disease, diabetes has been recognized by the United Nations as one of the four major non-communicable diseases. According to the International Diabetes Federation (IDF), an estimated 537 million adults worldwide are living with type 2 diabetes mellitus (T2DM) [1]. The widespread and severe complications of T2DM not only have a profound impact on individual health status, but also significantly increase the economic burden on national healthcare systems, making it one of the key issues to be addressed in the global public health arena [2-4].

Mild cognitive impairment (MCI) is a transitional stage between normal aging and dementia [5]. Patients often experience symptoms, such as communication impairment and memory loss. Research has consistently shown that diabetes mellitus is an independent risk factor for mild cognitive impairment, with diabetic patients experiencing an earlier onset and higher prevalence of cognitive dysfunction compared to those without the condition [6, 7]. Approximately 60-70% of people with T2DM have mild to moderate cognitive dysfunction [8]. Therefore, studying the relationship between diabetes and MCI is essential for understanding the mechanisms linking the two conditions.

Hypoglycemia, a common adverse event in the management of diabetes, is a major concern for patients with T2DM. Approximately 30-40% of insulin-treated T2DM patients experience at least one mild hypoglycemic episode each year [9, 10]. Impaired awareness of hypoglycemia (IAH) is defined as the occurrence of hypoglycemia with diminished sympathetic excitatory strength and/or symptoms of brain dysfunction [11]. IAH has an incidence of 8%-34.6% in patients with T2DM [12-14], and it is considered a risk factor for severe hypoglycemic events. IAH is associated with impaired learning and memory, with hypoglycemia contributing to the relationship between IAH and impaired cognition [15, 16]. Gold *et al.* found that diabetic patients with IAH exhibited more severe cognitive dysfunction than those without [17], which was confirmed by structural brain differences linked to cognitive function observed through magnetic resonance imaging [18]. Increasing evidence suggests that IAH contributed significantly to the development of SH and cognitive dysfunction in diabetic patients, though the exact mechanism remains unclear [19, 20]. Therefore, it is crucial to prioritize IAH in diabetic patients and address its adverse effects on cognitive impairment early.

Illness perception (IP) is defined as an individual's cognitive and affective representation of their illness [21]. It guides how individuals understand and perceive their condition, resulting in varied coping behaviors and emotional responses that lead to different clinical outcomes [22]. Existing studies have shown that IP is a predictor of quality of life in patients with MCI [23]. A positive IP enhances glucose control and delays the onset of mild cognitive impairment in T2DM patients [24, 25]. However, IAH has been reported to significantly impair patients' perception of their illness [26]. Given the relationship among IAH, IP, and MCI, we aimed to determine whether IP mediates the effect of IAH on MCI in T2DM patients.

Fear of hypoglycemia (FOH) refers to the negative emotional experience of behavioral change due to the threat of adverse consequences of hypoglycemia in the course of disease management, mainly in the form of psychological anxiety and behavioral changes [27]. Nearly half of people with T2DM who have experienced hypoglycemia develop FOH [28]. Studies have shown that FOH often leads patients to maintain higher blood glucose levels to avoid hypoglycemic episodes, resulting in elevated HbA1c levels that are linked to an increased risk of cognitive decline, including MCI [29]. Fariba *et al.* [30] found a significant positive correlation between IP and FOH in T2DM patients, with IP being one of the key factors influencing FOH. IAH is associated with more severe hypoglycemic episodes and greater FOH in patients with diabetes [31, 32]. In summary, we hypothesize that in T2DM patients, IP may influence FOH, which could serve as a potential mediator between IAH and MCI.

Deficits in illness perception among patients with IAH may potentiate the development of FOH [31, 32]. To cope with this fear, they frequently adopt compensatory behaviors to avoid hypoglycemic episodes, such as deliberately maintaining higher blood-glucose levels or reducing insulin doses [33]. Although these strategies may avert mild hypoglycemia in the short term, the resulting glycemic variability and persistent hyperglycemia can paradoxically increase the likelihood of unpredictable and more severe hypoglycemic events, thereby reinforcing fear and maladaptive behaviors and ultimately contributing to deterioration in cognitive function [34].

The common sense model (CSM) elucidates the relationships among illness perceptions, coping mechanisms, and health outcomes, delineating the manner in which individuals conceptualize and experience health threats and their strategies for coping with them [22]. In patients with T2DM, IAH manifests as a blunting or loss of symptom awareness, introducing substantial unpredictability into patients’ daily lives [35]. Psychological research indicates that living under such chronic uncertainty is a potent source of anxiety and emotional distress that undermines perceived control over illness-related events [36]. IAH, therefore, constitutes a multifaceted psychological threat that activates the symptom-representation processes described by the CSM. The onset of IAH alters patients’ illness perceptions [26], while the persistent and unpredictable nature of the threat engenders strong affective representations dominated by fear and anxiety [31, 32]. The CSM further posits a dynamic interaction between cognitive and emotional representations: negative illness cognitions amplify fear and promote maladaptive coping behaviors [22]. Consequently, the threat posed by IAH systematically modifies IP and increases FOH, both of which contribute to adverse health outcomes, such as MCI. Drawing on these CSM principles, we constructed the mediation model illustrated in Fig. (**1**). In summary, this study applied the CSM to examine the interplay among perception, coping mechanisms, and outcomes in T2DM patients with IAH.

## 2 METHODS

### 2.1 Study Design

Using convenience sampling, we enrolled patients with T2DM at two tertiary hospitals in Yangzhou, Jiangsu Province, China, from February to October 2022. These hospitals were selected for their large diabetes patient populations and their accessibility for participant recruitment and study implementation. The inclusion criteria for this study were as follows: (1) a confirmed diagnosis of T2DM for at least one year; (2) a history of hypoglycemia within the past six months; (3) age 18 years or older; (4) willingness to participate in the study; and (5) adequate communication and verbal abilities. Exclusion criteria included the following: (1) visual impairment due to complications or comorbidities; (2) severe psychiatric disorders; and (3) inability to read or write in Chinese.

As a cross-sectional study, we used G*Power 3.1.9.2 to calculate the required sample size for testing the mediation model. With an effect size of 0.15, a statistical power of 0.95, and a significance level (α) of 0.05 [37], the estimated minimum sample size was 129 participants. Accordingly, 275 questionnaires were distributed, and 251 were completed validly, yielding a 91.3% effective response rate. Ethical approval was granted by the Yangzhou University’s ethics committee (YZUHL20210022), and the study was conducted in accordance with the Declaration of Helsinki. All participants were informed of the study’s objectives, procedures, risks, and benefits, and they provided voluntary, written informed consent prior to enrollment.

### 2.2 Measurement Instruments

#### 2.2.1 General Information Questionnaire

The general information questionnaire collected data on gender, age, occupation, and marital status, while the medical information section assessed T2DM duration, comorbidities, complications, therapeutic regimen, and the number of hypoglycemic events.

#### 2.2.2 The Clarke Questionnaire

The eight-item questionnaire evaluated participants’ hypoglycemia history and their self-reported blood-glucose thresholds for symptom awareness. Responses were categorized as reduced awareness (R) or intact awareness (A), endorsing R on four or more items defined IAH. The Cronbach's α was 0.802 in our study [11].

#### 2.2.3 **The Hypoglycemia Fear Survey-Worry Subscale (HFS-WS**)

HFS-WS is a subscale of HFS used to measure hypoglycemia-related fears [38, 39]. The HFS-WS comprises 13 items, each rated from 0 to 4. Scores are summed from a total range of 0-52, with higher totals reflecting greater fear of hypoglycemia. The Cronbach's α was 0.921 in the study.

#### 2.2.4 The Brief Illness Perception Questionnaire (BIPQ)

The BIPQ is a nine-item instrument assessing cognitive and emotional illness perceptions [40]. The first eight items are rated on an 11-point Likert scale (0-10), with higher scores indicating more negative illness perceptions. The Cronbach's α was 0.710 in the Chinese version of the BIPQ [41].

#### 2.2.5 The Montreal Cognitive Assessment (MoCA) Questionnaire

The MoCA covers seven domains, including visuospatial/executive, naming, attention, language fluency, abstraction, delayed recall, and orientation, and produces a 0-30 score (higher scores reflect better cognition) [42]. One bonus point is added for fewer than 12 years of education, and a total ≥ 26 denotes normal cognitive function. Cronbach's α was 0.807 in the Chinese version of MoCA [43].

### 2.3 Statistical Analysis

Prior to data analysis, we assessed dataset completeness. Because the proportion of missing data for key variables was minimal (<5%), we employed listwise deletion and conducted the final analyses only on cases with complete data for all variables. Continuous variables have been expressed as mean ± SD (normal distribution) or median (IQR) (non-normal), with Pearson’s correlation for normally distributed variables and Spearman’s rank for non-normal variables. A two-tailed *p* < 0.05 denoted statistical significance. Mediation analysis was conducted in AMOS 27.0 using the bootstrap method (95% bias-corrected confidence interval). If the upper and lower limits did not include 0, it was deemed statistically significant.

## 3 RESULTS

### 3.1 Participant Characteristics

A total of 251 patients with T2DM participated in this study. Among these patients, 10.4% (n=26) had IAH. The sample included 62.2% females (n = 156), with 55.8% of the participants aged ≥ 60 years (n = 140). In terms of marital status, 93.2% (n=234) were married. Retirees accounted for the largest occupational category at 38.6% (n = 97). Disease duration ranged from 2 to 31 years, with 35.5% (n = 89) having had diabetes for less than 10 years. Half of the patients (50.2%, n = 126) reported one to two diabetic complications, most commonly hypertension and hyperlipidemia. With respect to the frequency of hypoglycemia in the past six months, 76.1% (n = 191) of patients experienced it one to two times. Table **1** shows the detailed sociodemographic and clinical characteristics of patients with T2DM.

### 3.2 The Scores of each Scale in T2DM Patients

Among patients with IAH, the mean Clarke score was 1.65 ± 1.54 (range 0-6), indicating substantial inter-individual variability in impaired hypoglycemic awareness. The mean HFS-WS score was 16.35 ± 5.25 (range 9-39), reflecting a moderate level of fear of hypoglycemia with wide individual differences. The mean BIPQ score was 37.74 ± 5.89 (range 24-60), indicating a moderate level of illness perception. The mean MoCA score was 26.90 ± 3.10 (range 19-30); 99 patients (39.4%) scored below 26, consistent with MCI. The results of the descriptive statistical analysis of the variable scores are shown in Fig. (**2**).

### 3.3 Association among IAH, FOH, IP, and MCI

Table **2** shows the relationships among IAH, FOH, IP, and MCI. The results demonstrated that the variables correlated with varying degrees, and that these differences were statistically significant (*p* < 0.01). IAH, FOH, and IP were negatively related to MCI (r=-0.333 for IAH, r=-0.244 for FOH, r=-0.260 for IP). IAH was positively correlated with FOH (r =0.336). Both IAH and FOH were also positively correlated with IP (r = 0.307 and r = 0.353, respectively).

### 3.4 Association between IAH and MCI and the Mediating Effects of IP and FOH

The mediated effects model showed good overall model fit, and no adjustments were needed Fig. (**3**) and Table **3**. IAH negatively predicted MCI with an effect size of -0.40. Both IP and FOH also negatively predicted MCI with effect sizes of -0.17 and -0.28, respectively.

IAH exerted a significant direct effect on MCI, accounting for 40.1% of the total effect (95% CI excluding zero), indicating a significant impact. The total indirect effect accounted for 29.3% of the total effect size. The total indirect effect consisted of 3 pathways. First, IAH affected the MCI through IP, and this pathway accounted for 9.1% of the total effect size. Second, IAH affected the MCI through FOH, and this pathway accounted for 15.4% of the total effect. Finally, IP and FOH acted as chain mediators in the association between IAH and MCI, and this pathway accounted for 4.8% of the total effect size (Table **4**).

## 4 DISCUSSION

Clarke questionnaire score was negatively correlated with the MoCA score (*p* < 0.01), indicating that more severe impairment of hypoglycemic awareness increases the likelihood of mild cognitive impairment, consistent with previous follow-up studies in Edinburgh samples [20]. Evidence from neuroimaging studies indicates that, compared to individuals with intact hypoglycemia awareness, patients with IAH exhibit reductions in gray-matter volume and cortical surface area in frontal and parietal regions [18]. Functional imaging has also shown that, in patients with IAH, brain regions involved in arousal, decision-making, and reward processing exhibit altered responses to hypoglycemia [44]. Together, these structural and functional abnormalities likely contribute to the cognitive decline observed in patients with IAH. Patients with IAH have a six-fold higher risk of SH compared to those with intact awareness [45]. SH can provoke significant stress and anxiety in patients, which can affect self-management and glycemic control. Lacy *et al.* [46] demonstrated SH to be associated with cognitive decline, particularly in memory and executive function. Hypoglycemia, *via* mechanisms, such as altered cerebral blood-flow distribution, induces cumulative neuronal injury in metabolically active brain regions [47]. Over time, these cellular-level insults evolve into macroscopic cerebral atrophy, thereby increasing the risk of MCI [48]. Therefore, healthcare professionals should focus on preventing SH to mitigate the impact of IAH on MCI and support cognitive health. This can be achieved through patient education on symptom recognition, more frequent blood-glucose monitoring, and the use of continuous glucose monitoring systems that provide real-time alerts.

The findings indicated that IP partially mediates the relationship between IAH and MCI. Relevant studies have suggested IP as a predictor of quality of life in patients with MCI [23, 49]. Specifically, Kim *et al.* [49] demonstrated a strong association between IP and MCI in patients with T2DM. Moreover, several studies have reported a relationship among IP, glycemic control, and MCI in patients with T2DM. These studies show that a positive IP is essential for maintaining glycemic control and potentially mitigating cognitive decline [24, 25]. In contrast, patients with IAH are prone to more frequent hypoglycemic episodes, which can elicit negative emotions and lead some to adjust their insulin dosing to avoid further low levels, thereby reinforcing negative illness perceptions. This cycle, therefore, places patients with IAH at greater risk for MCI. These results suggest that healthcare providers should prioritize interventions that enhance patients’ illness perceptions to reduce the likelihood or severity of cognitive impairment.

IAH was observed to affect MCI through the partial mediating effect of FOH. Our study showed a significant correlation between IAH and FOH. Consistent with these findings, Habte-Asres *et al.* [50] found that patients with IAH undergoing hemodialysis had more severe hypoglycemic fear than patients without IAH. Similarly, Lin *et al.* [51] also found the same results in a qualitative study involving IAH patients. These results underscore the strong association between IAH and FOH. Additionally, a significant negative correlation was found between FOH and MCI, indicating that patients with more severe FOH were more likely to experience MCI. Patients with FOH often experience negative emotions, such as anxiety and depression [52, 53]. Several studies have reported that anxiety increases the risk of MCI [54, 55]. Furthermore, most patients with FOH have been reported to experience hypoglycemic episodes, which can adversely affect multiple aspects of quality of life in T2DM patients, including emotional well-being, behavior, cognition, and social functioning [56]. Therefore, healthcare providers should assess and manage FOH in T2DM patients with IAH, reducing MCI risk through psychological counselling to ease fear and enhance coping.

IP and FOH played a chain mediating role in the relationship between IAH and MCI. The results of the study found that IP positively predicted FOH. When T2DM patients develop IAH, they often struggle to recognize symptoms of hypoglycemia, leading to changes in their cognitive and emotional representations of the illness [57]. This, in turn, prompts behavioral changes aimed at avoiding hypoglycemia, ultimately contributing to the development of FOH. Patients with T2DM often adopt avoidance behaviors to prevent hypoglycemia, for instance, by reducing insulin dosage or increasing food intake [58]. These actions, however, can result in inadequate glycemic control, which exacerbates their condition and accelerates the onset of related complications, thereby potentially accelerating the development of MCI.

Thus, in clinical practice, healthcare professionals should fully consider the interrelationships among IAH, IP, FOH, and MCI. Clinicians should promptly identify patients with IAH and deliver targeted psychoeducation grounded in the CSM to effectively modify negative illness perceptions [26, 59]. Interventions, such as cognitive behavioral therapy [60], together with glucose-management technologies, including continuous glucose monitoring (CGM) [61] and automated insulin delivery systems [62], can reduce FOH, and may help prevent or mitigate the risk of MCI.

## CONCLUSION

Our study found that the association between IAH and MCI was mediated by IP and FOH. This suggests that healthcare professionals could prevent MCI in patients with T2DM by considering interventions for both IP and FOH. On one hand, healthcare professionals should focus on assessing and improving IP in individuals with T2DM, particularly those with IAH. This can be achieved by enhancing patients’ understanding of hypoglycemia and correcting negative illness beliefs, thereby reducing the risk of cognitive decline. On the other hand, they should also work to mitigate FOH through psychological interventions, such as counseling or cognitive-behavioral therapy, in IAH patients, which can help to indirectly lower the likelihood of MCI in this population.

This study was limited by its setting in only two hospitals in China and its reliance on self-reported data. The cross-sectional design prevented causal analysis. Future large-scale, longitudinal studies using objective measures, like CGM, are needed to confirm these findings.

## Figures and Tables

**Fig. (1):**
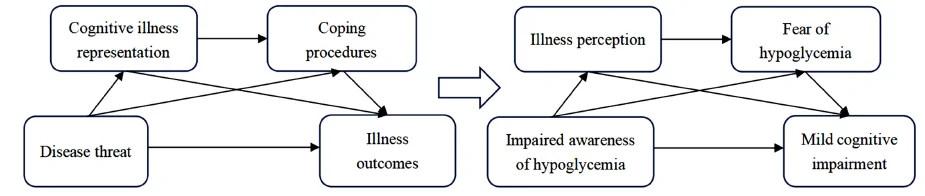
The relationship among illness perceptions, coping mechanisms, and health outcomes: a hypothesis model.

**Fig. (2):**
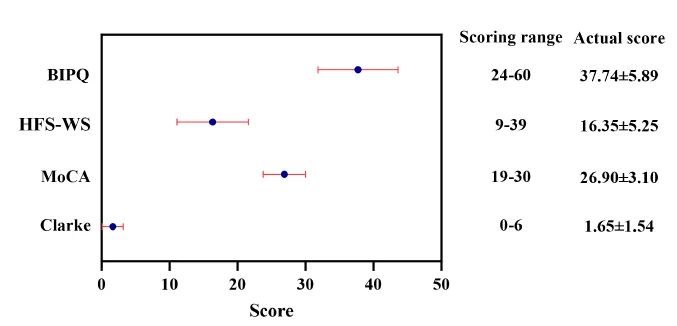
Descriptive statistical analysis of variable scores. BIPQ: Brief Illness Perception Questionnaire, HFS-WS: Hypoglycemia Fear Survey-Worry Subscale, MoCA: Montreal Cognitive Assessment.

**Fig. (3):**
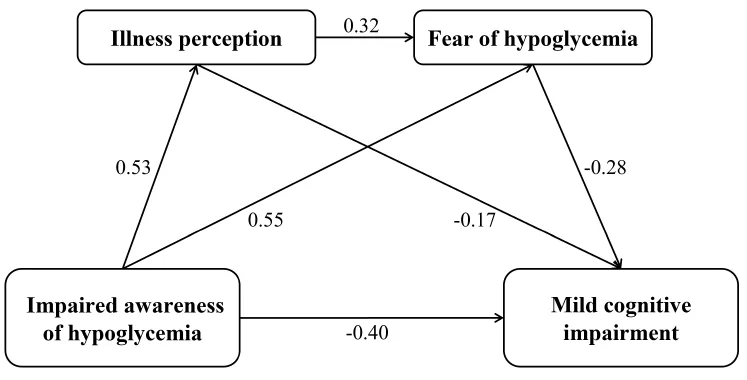
Serial mediation models for IAH, IP, FOH, and MCI in persons with T2DM.

**Table 1:** Characteristics of patients with T2DM (n=251).

**Variables**	**Number**	**Percentage (%)**
**Gender**	-	-
Male	156	62.2
Female	95	37.8
**Age (years)**	-	-
18-44	25	10.0
45-59	86	34.3
≥60	140	55.8
**Occupation**	-	-
On job	84	33.5
Retired	97	38.6
Farmer	19	7.6
Other	51	20.3
**Marriage**	-	-
Married	234	93.2
Unmarried	6	2.4
Divorced	3	1.2
Widowed	6	3.2
**Duration of diabetes (years)**	-	-
<10	89	35.5
10-19	96	38.2
≥20	66	26.3
**Complications**	-	-
0	113	45.0
1-2	126	50.2
>2	12	4.8
**Comorbidities**	-	-
0	56	22.3
1-2	138	55.0
>2	57	22.7
**Treatment**	-	-
None	3	1.2
Oral medication	71	28.3
Insulin shots	116	46.2
Oral medication together with insulin shots	61	24.3
**Hypoglycemia frequency in the recent 6 months**	-	-
1-2	191	76.1
3-6	45	17.9
>6	15	6.0

**Table 2:** Correlation among IAH, FOH, IP, and MCI in persons with T2DM (n=251).

**Variables**	**IAH**	**FOH**	**IP**	**MCI**
IAH	1			
FOH	0.336**	1		
IP	0.307**	0.353**	1	
MCI	-0.333**	-0.244**	-0.260**	1

**Abbreviations: ** IAH: Impaired awareness of hypoglycemia; FOH: Fear of hypoglycemia; IP: Illness perception; MCI: Mild cognitive impairment; ** *p*<0.01.

**Table 3:** Goodness of fit of the model.

**Index**	**χ^2^/df**	**GFI**	**NFI**	**CFI**	**IFI**	**AGFI**	**RMSEA**
Model	2.657	0.993	0.943	0.942	0.964	0.938	0.074
Criterion	<3	>0.9	>0.7	>0.9	>0.9	>0.9	<0.08

**Abbreviations: **χ^2^/df =Chi-square to degrees of freedom ratio; GFI = Goodness-of-fit index; NFI = Normed fit index; CFI = Comparative fit index; IFI = Incremental fit index; AGFI = Adjusted goodness-of-fit index; RMSEA = Root mean square error of approximation.

**Table 4:** The path coefficient of the mediated effect model (n=251).

**Route**	**Effect value**	**S.E.**	**Boot LLCI**	**Boot ULCI**
Route 1:IAH→IP→MCI	-0.091	0.049	-0.126	-0.056
Route 2:IAH→FOH→MCI	-0.154	0.022	-0.172	-0.102
Route 3:IAH→IP→FOH→MCI	-0.048	0.023	-0.062	-0.035
Total effect	-0.293	0.507	-0.338	-0.248
Indirect effect	-0.401			

**Abbreviations: **IAH: Impaired awareness of hypoglycemia; FOH: Fear of hypoglycemia; IP: Illness perception; MCI: Mild cognitive impairment; S.E.: Standard error; Boot LLCI: Bootstrap 95% lower limit confidence interval; Boot ULCI: Bootstrap 95% upper limit confidence interval.

## Data Availability

All the data and materials used in this study will be made available by the corresponding author on reasonable request.

## LIST OF ABBREVIATIONS

IDF: International Diabetes Federation
T2DM: Type 2 Diabetes Mellitus
MCI: Mild Cognitive Impairment
SH: Severe Hypoglycemia
IAH: Impaired Awareness of Hypoglycemia
IP: Illness Perception
FOH: Fear of Hypoglycemia
CSM: Common Sense Model
HFS-WS: Hypoglycemia Fear Survey-Worry Subscale
BIPQ: Brief Illness Perception Questionnaire
MoCA: Montreal Cognitive Assessment
CGM: Continuous glucose monitoring

## STANDARDS OF REPORTING

STROBE guidelines were followed.
